# Integrating eHealth data analytics into Continuing Professional Development for medical practitioners: an ecosystemic perspective

**DOI:** 10.3389/fmed.2025.1553479

**Published:** 2025-03-28

**Authors:** Carol Pizzuti

**Affiliations:** Department of Medical Education, Melbourne Medical School, The University of Melbourne, Parkville, VIC, Australia

**Keywords:** Çontinuing Professional Development (CPD), eHealth data analytics, medical practitioners, ecosystemic perspective, CPD ecosystem

## Abstract

This perspective explores the evolving landscape of Continuing Professional Development (CPD) for medical practitioners, focusing on the use of eHealth data analytics to strengthen CPD programs and practices. Traditional didactic approaches to CPD have demonstrated limitations, prompting a shift toward outcome-focused and workplace-based CPD activities. This trend aligns with medical regulations that emphasize integrating clinical performance and patient health data into professional learning for practice change and improved care. Leveraging eHealth data analytics for self-assessment, improved clinical performance, and effective CPD is emerging as an opportunity. Both academia and industry are actively working to link clinical performance data, continuous learning, and CPD to promote safer, higher-quality care. eHealth data analytics enables personalized CPD by addressing specific performance gaps and clinical needs, enhancing learning impact and health outcomes. However, current research highlights challenges such as data accessibility, availability, and quality, technological interoperability, and resistance to change—both organizationally and at the individual level. These obstacles underscore the need for a holistic approach, innovative thinking, and evidence-based solutions in the ever-changing fields of medical regulation and continuing education. Further research is essential to substantiate the value of eHealth data for CPD, build a comprehensive depiction of the CPD ecosystem, and guide successful implementation and cultural shifts. Building a data-driven CPD ecosystem requires interdisciplinary collaboration and a commitment to real-world solutions. Future efforts must focus on both theoretical and applied exploration to fully realize the value of eHealth data analytics, enabling personalized, impactful CPD in a fast-moving healthcare environment.

## Introduction

Over the past two decades, there has been a slow yet steady shift toward strengthening Continuing Professional Development (CPD) for medical practitioners (1–5).

It has been widely acknowledged that conventional didactic and educational CPD activities (also called CME, Continuing Medical Education) have limited effectiveness in enhancing clinical practice and improving patient outcomes (6–8). Moreover, during this time, a consensus has emerged among health professionals’ educators on the value of CPD activities that promote the use of external assessment and feedback for self-reflection on practice and behavior change (9–12).

As part of this ongoing shift, some current debates in the field focus on the evolving role of CPD and its integration into broader healthcare systems and medical education principles and practices. A key aspect of these discussions highlights the need to anchor CPD within real-world professional environments, emphasizing the importance of workplace and practice settings in facilitating meaningful learning and the acquisition of professional competencies (13–15). Another major focus is the application of the Competency-Based Medical Education (CBME) paradigm (16, 17) and Entrustable Professional Activities (EPAs) frameworks (18) to CPD, aiming to enhance patient care and outcomes while building continuity of assessment and continuous improvement from undergraduate and postgraduate medical education into CPD (19–21).

In response to these developments, more emphasis is currently being given to CPD activities related to daily practice (22), aligned with professional standards (21) and workplace assessment (23, 24), and based on health outcomes measurement (25, 26) and quality improvement (19, 27).

Both academia and industry are presently engaged in research to strengthen the linkage between clinical performance data, medical practitioners’ learning and CPD, and practice change (28–38). Concurrently, a number of medical regulatory bodies have recently launched CPD policies aimed at strengthening CPD through:

- the development of CPD programs more aligned to practitioners’ scope of practice and clearly interrelated to quality care and patient safety (39–43); and
- a focus on workplace based CPD activities that require the use of clinical performance data and patient health data analytics, such as Audit and Feedback (A&F) interventions, Quality Improvement (QI) projects, and Mortality and Morbidity Meetings (MMM) (44–47).

To achieve these goals, it is also crucial to recognize the fundamental role and needs of both patients and medical practitioners, ensuring that their perspectives and lived experiences will shape the evolution of CPD with the ultimate aim of effectively supporting the delivery of safer and higher-quality care.

In this context, the use of eHealth data analytics to support self-reflection on clinical practice, promote performance improvement, and strengthen CPD represents an opportunity that should not be underestimated. eHealth data analytics could enhance workplace learning by identifying specific areas for improvement and fostering a culture of continuous professional development within practice settings. Moreover, it would support the effective implementation of CBME and EPAs in CPD by providing real-time outcome data and enabling personalized feedback, ensuring that CPD activities remain relevant and aligned with the competencies required in contemporary healthcare.

In light of the lack of a universally accepted definition and to maintain a comprehensive scope, this perspective uses the term “eHealth data” to encompass any personal information in digital or electronic format that relates to an individual’s health status, risks, or outcomes, as well as data associated with the delivery of healthcare services and interventions (48–51). Given this, “eHealth data” broadly includes various types of health-related digital records, such as Electronic Medical Records (EMRs), Electronic Health Records (EHRs), registries, routinely collected administrative data, claim and billing data, electronic prescriptions, Patient-Generated Health Data (PGHD), Patient-Reported Outcome Measures (PROMs), and Patient-Reported Experience Measures (PREMs).

A key challenge for this emerging field is that it operates in a pre-existing complex environment–the “CPD landscape” or “CPD ecosystem.” According to international medical educators, the CPD ecosystem is made of several stakeholders, i.e., medical practitioners, patients, health professions education academics and researchers, medical regulators and policy-makers, CPD providers and educators, healthcare service organizations and health care systems’ leaders (52). Also, there is agreement on the collective responsibility and action of all stakeholders to strengthen CPD for improved practice and safer care (53).

Despite this, there is little research on how these stakeholders operate and interact within the whole CPD ecosystem (54). In particular, there is no previous research on digital health innovation and implementation within the CPD ecosystem.

In order to address this gap, this perspective examines the roles and interrelationships of key stakeholders in the CPD ecosystem, specifically exploring how their functions and contributions can foster the integration of eHealth data analytics to strengthen CPD. The goal is to propose a more holistic, interconnected approach to CPD research, governance, and practice.

## eHealth data analytics and CPD: an ecosystemic perspective

**Figure 1:**
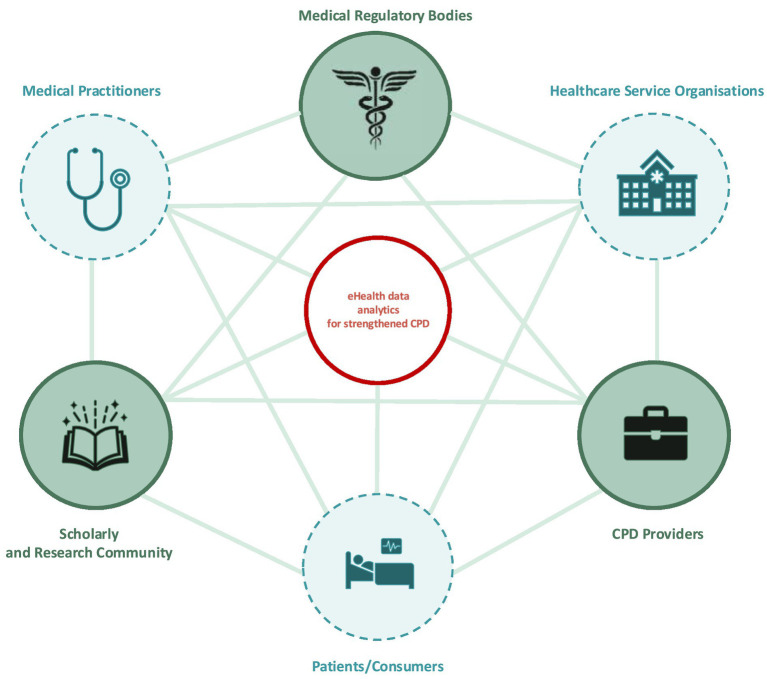
CPD ecosystem.

**Figure 2:**
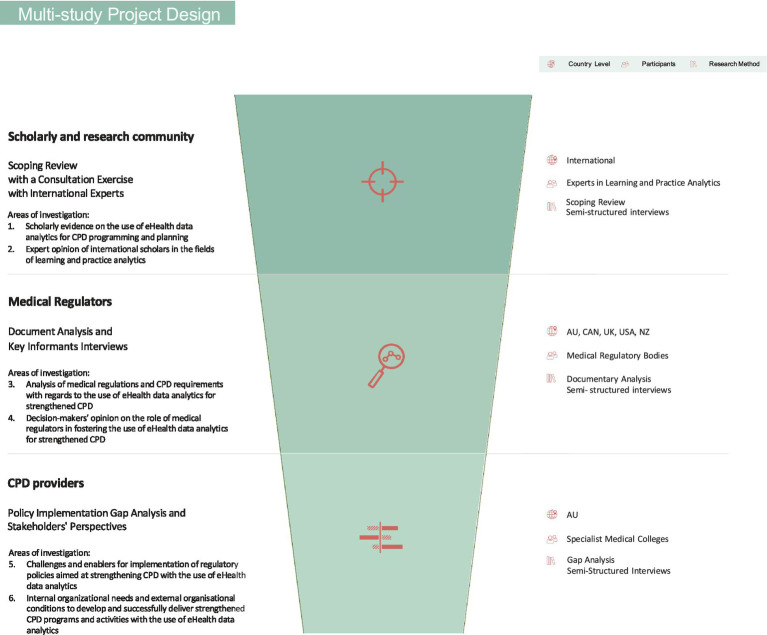
Design of the multi-study project titled “Using eHealth data to strengthen Continuing Professional Development (CPD) for medical practitioners: an exploration of regulatory and organizational factors influencing eHealth data analytics implementations within the CPD ecosystem”.

The insights presented in this perspective are drawn from the integrated findings of a multi-study project titled “Using eHealth data to strengthen Continuing Professional Development (CPD) for medical practitioners: an exploration of regulatory and organizational factors influencing eHealth data analytics implementations within the CPD ecosystem” (55), which examined the roles of three key stakeholders in the CPD ecosystem: the scholarly and research community, medical regulators, and CPD providers (illustrated in the green circles in Figure 1). This project consisted of three interrelated studies (56–58), contributing to the exploration of six distinct areas of investigation across targeted jurisdictional settings (Figure 2). These areas of focus provide a structured framework for analyzing how each stakeholder contributes to promoting the integration of eHealth data analytics into CPD.

While the primary focus of this project was on three central stakeholders, the research also offers valuable insights into the roles of additional stakeholders within the broader CPD ecosystem. These include healthcare organizations, patients, and other actors whose involvement plays a crucial role in the successful implementation and adoption of eHealth data analytics in CPD.

## Scholarly and research community: value and opportunity vs. uncertainty

Most CPD stakeholders acknowledge the value of and the opportunity in using eHealth data analytics for CPD. This recognition aligns with scholarly research, which presents recommendations for linking clinical performance data (including eHealth data) and workplace-based assessment with continuous learning, CPD, and practice change (21, 23, 28, 59, 60). Moreover, a body of literature exists on the exploration of current ecosystemic and cultural factors that might influence data-driven CPD practices (30–32, 37, 61, 62) and on potential implementation of eHealth data analytics technologies to strengthen CPD (35, 36, 63).

However, it is worth noting that these research outputs are largely speculative or in their infancy. In addition, latest research findings in this space emphasize how practical implementation of eHealth data analytics use for CPD poses significant challenges (7, 56), describing this “theory-to-practice challenge as a potential conceptual misstep” (33) (p. 20). These developments would suggest that the concepts of “value” and “opportunity” in using eHealth data analytics for CPD are mainly grounded in theoretical principles rather than being substantiated by robust applied research (33).

This existing gap between theory and practice holds significant implications. First, the scarcity of practical evidence introduces uncertainty regarding the effectiveness of the use of eHealth data analytics for CPD, and could potentially lead to a conservative approach with regards to regulatory support, industry adoption, and medical practitioners’ acceptance. Second, policymakers and industry CPD stakeholders may exercise caution in promoting the use of eHealth data analytics for CPD and investing in its implementation due to the absence of evidence supporting its benefits for practice improvement and better patient outcomes.

These implications underscore the pressing need to bridge the divide between theory and practice in eHealth data analytics for CPD, as it is crucial to enable informed decision-making and to ensure strategic investments that harness the full potential of this field.

On top of this reflection, it has to be noted that, in addition to the gap between theory and practice, significant uncertainty remains regarding both the theoretical foundations and practical applications of eHealth data analytics for CPD. Findings from Pizzuti et al. (56, 64) detail a lack of agreement on both underlying conceptualizations and real-world operationalization of eHealth data analytics principles and technologies for strengthened CPD. Interestingly, the industry sector—including medical regulators and CPD providers— appears to have an active interest in the use of eHealth data analytics for CPD, even though accompanied by concerns around conceptual and theoretical underpinnings, as well as meaningful, feasible, and effective application.

A critical point of uncertainty is the conceptual ambiguity surrounding the term “performance data.” This ambiguity appears to hinder effective communication among stakeholders and is delaying the successful implementation of eHealth data analytics for CPD in real-world scenarios.

According to Tavares et al. (33), the umbrella term “performance data” is used to encompass both feedback and patient health data, though questions remain about whether this term is the correct conceptual linchpin for using workplace-based assessment for learning and CPD. Furthermore, findings from Pizzuti et al. (57) show that this conceptual uncertainty is also present in current regulatory policies and CPD requirements for medical practitioners. Regulators view “performance data” as not only encompassing eHealth data analytics’ outcomes, but also results from colleagues’ and patients’ questionnaire derived from Multi-Source Feedback (MSF) tools, as well as assessments from peers or non-physician observers during practice visits.

This lack of distinction between *patient health data-based CPD activities* and *feedback-based CPD activities* creates potential for miscommunication among CPD stakeholders, especially between scholars and researchers, and industry professionals in specialized domains like digital health, data science, and eHealth data management.

The focus in these specialized fields has predominantly been on leveraging patient eHealth data and digital technologies to enhance clinical practice and healthcare (64, 65), not on the use of MSF tools, the analysis of colleagues’ and patients’ questionnaire results, or the documentation resulting from practice visits. As a result, the lack of distinction between these types of data can cause misalignment in research aims, outcomes, and expectations for the future use of eHealth data analytics technologies in CPD.

Further support for these findings comes from Pizzuti et al. (58), where CPD providers report that the lack of differentiation between *feedback-based* and *patient health data-based* CPD activities presents a significant barrier to the full implementation of regulatory policies aimed at strengthening CPD with the use of eHealth data analytics.

In light of these issues, it is clear that more research is needed to resolve the ambiguity surrounding the term “performance data” and to propose a more precise conceptual framework. Collaboration between scholars, regulatory bodies, and CPD providers is essential to create common ground, share knowledge, and develop effective definitions that will support the implementation of eHealth data analytics in the CPD ecosystem.

## Medical regulatory bodies: current policy content, regulators’ potential role, and future regulatory policy development

According to Pizzuti et al. (57), the *content* of current CPD requirements revolves around two main concepts:

- The broad conceptualization of the term “data” as any piece of information pertaining to medical practitioners’ practice and/or performance–thus including patients’ and colleagues’ feedback, and not only patient health data.
- The acknowledgement of eHealth data as a *potential* data source for CPD completion and compliance.

These aspects stem directly from the institutional *role* of medical regulators in the CPD ecosystem (66–69) and their *current stance* on eHealth data use (57):

- Regulatory bodies offer overarching guidance concerning CPD requirements.
- While acknowledging the potential of eHealth data analytics to strengthen CPD, regulators perceive its exclusive use for performance assessment purposes as limiting, supporting a more diversified data approach.

Given this perspective, the current discretionary use eHealth data for CPD will likely persist unless regulators reassess their own role and responsibilities within the CPD ecosystem—particularly concerning CPD requirements. A more proactive stance on the use of eHealth data analytics for CPD, alongside policy development and implementation, is necessary for its effective integration.

In light of this, medical regulators are called to assume a leadership role in the CPD ecosystem to address the barriers to adopting eHealth data analytics for CPD (57). Key responsibilities include advocating for eHealth data usage, collaborating with stakeholders such as governments, healthcare sectors, and organizations responsible for eHealth data management, and establishing partnerships with data experts and research groups to integrate eHealth data into medical regulatory processes.

When it comes to policy development, universally applicable recommendations for medical regulators is challenging due to the need for further research on key policy concepts, as well as gaps in understanding of organizational culture, vision, legislative environments, and jurisdictional variances (57).

Despite these challenges, the following recommendations can guide policy decision-making for medical regulatory bodies:

- Develop and incorporate a precise definition for critical terms such as “data” in regulatory policies and related supporting documents.
- Clearly articulate policy intentions concerning regulators’ expectations for eHealth data utilization and analysis, as well as their plans for future policy development and implementation within the CPD ecosystem.

The first recommendation carries specific weight due to the current ambiguity surrounding the term “performance data,” as discussed above. The second recommendation is crucial for creating clear regulatory policies and transparent communication of intent, which can open opportunities for regulators to:

- Contribute to the ongoing discourse on strategies centered on performance-based assessment and data-driven CPD (25).
- Promote digital health innovation within CPD practices.
- Clarify the formative use of eHealth data analytics and its educational benefits so to address concerns among medical practitioners regarding potential punitive actions in the case of poor performance outcomes.
- Encourage other key CPD stakeholders to engage in the current discussion, consider their own roles in advancing the use of eHealth data analytics for CPD, and assume responsibilities for supporting its implementation.

## Healthcare service organizations: data issues and business and legal priorities

All CPD stakeholders investigated in the multi-study project describe the existence of various data and/or data system issues within the CPD ecosystem, identifying them as the primary challenges inhibiting the adoption of eHealth data analytics for CPD (56, 57, 64). These findings confirm existing research documenting the less-than-optimal state of data quality and infrastructure in the health system (70–77) and align with recent scholarly insights by Tavares et al., who emphasizes “data infrastructure” as a pivotal but unresolved issue for leveraging eHealth data analytics in CPD (33). Specifically, Tavares et al. characterize “data infrastructure” as a “leap” in the field – an “underlying assumption that may be necessary for successful integration but may not yet be resolved” (33) (p. 13).

The integrated findings from the multi-study project also suggest that research participants consider “data issues” as pertaining to healthcare service organizations. Further investigation into this crucial CPD stakeholder is currently necessary. Future research in this area will not only deepen existing insights but also help formulate practical solutions to address the challenges surrounding data infrastructure.

In addition to these data challenges, a reflection on the findings of Pizzuti et al. (56) would suggest that the business and legal imperatives that govern the healthcare system and its operating framework are having a hindering effect on the implementation of eHealth data analytics in the CPD ecosystem. These imperatives, coupled with the absence of political and motivational drivers for using eHealth data analytics for CPD, reflect a limited focus on education and professional development at the healthcare service level.

In this context, establishing robust partnerships between healthcare service organizations and other CPD stakeholders—particularly CPD providers—proves challenging.

Further investigation is necessary to validate these considerations. Also, active involvement and contributions from healthcare service organizations are crucial to deepen these insights and to avoid inter-organizational silos in the CPD ecosystem.

## CPD providers: current challenges and future action

Currently, several internal organizational characteristics and a number of ecosystemic factors are hindering CPD providers’ efforts in implementing the use of eHealth data analytics for CPD (64).

At the organizational level, one significant issue is the allocation of internal resources to CPD offices. Despite advancements in educational technologies (78–80) and evidence on CPD cost-effectiveness (81, 82), many CPD providers still face resource constraints in managing and delivering CPD programs effectively. Furthermore, engagement with members and communication about CPD activities remain areas in need of improvement. The effectiveness of CPD programs in improving care quality and patient safety is another concern, with limited evaluation processes currently in place (22, 83). Additionally, accountability measures for CPD compliance are often insufficient, with international trends (84) showing an increasing focus on mandatory requirements such as appraisals (85) and annual conversations (40). Governance and approval processes, particularly in organizations that are predominantly self-regulated (i.e., specialist medical colleges and professional associations), also contribute to the complexities faced by CPD providers (64).

On the ecosystemic level, external factors such as data fragmentation, availability, and accessibility in the healthcare system (74) pose significant challenges. Among other issues, the lack of standardization of existing performance assessment and measuring outcomes tools and activities at healthcare service level (86, 87) and the immature feedback and reflective practices in medical education and practice (88, 89) hinder the effective implementation of eHealth data analytics for CPD.

Given the clear identification of the key challenges, urgent action and focused research are now essential to overcome these barriers and ensure the successful integration of eHealth data analytics into CPD practices.

## Medical practitioners’ attitudes and the cultural impact of medical self-regulation

The attitudes of medical practitioners toward CPD and the use of eHealth data analytics for CPD have been identified as significant challenges across the studies conducted in the multi-study project (56, 57, 64). This aligns with existing literature, which similarly highlights difficulties in engaging medical practitioners with CPD (90–92)—with many viewing it merely as a “tick-box” exercise (58, 93). Additionally, exploratory research reports the prevalent distrust among medical practitioners regarding eHealth data analytics, and their apprehensions about potential punitive uses of such data (31, 33, 37, 56, 58, 61).

Recent advancements in Health Professions Education (HPE) underscore the critical role of *trustworthiness* in the field of eHealth data analytics for CPD - considering it as another “theory-to practice leap” (33). Despite this, the issue of trust remains unresolved, with limited evidence or recommendations on how to address it effectively.

This gap represents a problem as medical practitioners have expressed interest in accessing routinely collected clinical data for education and professional development (37). This interest stems from the potential of data analytics results, which may enable clinicians to compare their performance with peers, encourage team reflective discussions, and support practice change (61).

In examining these challenges, two key factors emerge as central to the successful adoption of eHealth data analytics for CPD by medical practitioners. First, the current lack of robust evaluation processes to assess the impact of CPD activities on medical practice and quality of care (64) directly correlates with the negative attitudes that medical practitioners hold toward CPD. Without comprehensive assessment mechanisms, practitioners may remain skeptical about the value and impact of CPD activities. Second, the presence of multiple data issues at the healthcare service level contribute to medical practitioners’ distrust of eHealth data analytics. Addressing these limitations is crucial to fostering more positive attitudes toward data-driven CPD among medical practitioners.

Furthermore, there is a causal relationship between the cultural aspect of self-regulation, which characterizes the medical profession, and the implementation of eHealth practice analytics for strengthened CPD (57, 64). The professional culture within medicine has historically upheld self-regulation as a cornerstone (94), which in turn shapes how CPD is approached and influences the adoption of innovative methods, such as the use of eHealth data analytics.

Within this cultural framework, self-regulation not only aligns with but also significantly influences the scarcity of established accountability measures related to CPD compliance, particularly with regard to completing patient health data-based CPD activities (57). Moreover, self-regulation can reinforce the discretionary use of eHealth data analytics among medical practitioners due to their autonomy and professional independence. The absence of stringent guidelines or mandated requirements for utilizing eHealth data analytics in CPD (57) fosters an environment where practitioners have the choice to determine the extent and manner in which they engage with eHealth data and eHealth data analytics technologies.

A deeper investigation into the cultural aspects of the medical profession is essential to understanding how these factors influence the uptake of eHealth data analytics in CPD. Such research would provide critical insights into how cultural norms shape the integration of new technologies and practices in continuing medical education and professional development.

## Patients: a missing perspective

The integrated findings of the multi-study project (55–58) reveal a lack of recognition regarding patients’ importance, contribution, or role in the conceptualization and implementation of eHealth data analytics for strengthened CPD.

Some research evidence is available on patients’ insight on and involvement in medical practitioners’ CPD (95, 96), and on patients’ perspectives on the use of eHealth data for self-reflective practices (34). However, an extensive body of literature on these matters is currently lacking, indicating the need for more research in this space.

## eHealth data system vendors: key stakeholders in the CPD ecosystem

Insights gleaned from international experts in learning and practice analytics highlight the prevalence of proprietary approaches in data analytics technology (56). This underscores the pivotal role of eHealth data system vendors within the CPD ecosystem, and the necessity of positioning them as key stakeholders in CPD.

Recent research, exemplified by Pusic et al. (28), further supports this notion by advocating for the enhancement of existing health information systems to actively foster learning. The electronic health record, often underestimated as an educational tool, holds significant potential to elevate education and evidence-based healthcare.

Acknowledging this potential, vendors’ role, influence, and potential contributions to strengthened CPD through the use of eHealth data analytics should be thoroughly examined, and integrated into future decision-making and collaborative efforts aimed at advancing the use of eHealth data analytics for CPD.

## Conclusion

The integration of eHealth data analytics into CPD is widely regarded as a valuable opportunity to enhance medical practitioners’ professional development and to foster CPD activities linked to clinical practice. Despite its potential, implementing eHealth data in CPD presents several challenges, particularly due to the multifaceted nature of the issue, the involvement of diverse stakeholders, and uncertainties in practical application.

As highlighted throughout this perspective, the complex nature of this subject requires a holistic approach, innovative thinking, and collaboration across stakeholders, with a steadfast commitment to evidence-based and real-world solutions. Importantly, it will be critical to acknowledge and integrate the perspectives and contributions of patients, medical practitioners, and healthcare teams to ensure that the evolution of CPD through the use of eHealth data analytics remains relevant and effective.

The pursuit of leveraging eHealth data for strengthened CPD remains a work in progress, but one that is essential in the evolving landscape of medical regulation and continuing education for medical practitioners. Further research, both theoretical and applied, is necessary to substantiate the value of using eHealth data for CPD purposes, refine our understanding of the CPD ecosystem, and provide guidance for successful implementation and a meaningful cultural shift in CPD practices.

Looking ahead, the future success of eHealth data analytics in CPD will depend on the ability to promote communication, collaboration, and engagement among all stakeholders within the CPD ecosystem.

## Data Availability

The raw data supporting the conclusions of this article will be made available by the author, without undue reservation.
